# Utility of the Ratio of Adenosine Deaminase and Serum C-reactive Protein in Differentiating Parapneumonic, Tuberculous, and Malignant Pleural Effusions

**DOI:** 10.7759/cureus.65659

**Published:** 2024-07-29

**Authors:** Sachinkumar S Dole, Arun Balan, Nitin S Gaikwad

**Affiliations:** 1 Respiratory Medicine, Dr. D. Y. Patil Medical College, Hospital & Research Centre, Dr. D. Y. Patil Vidyapeeth, Pune, IND; 2 Respiratory Medicine, Maharashtra Institute of Medical Education and Research (MIMER) Medical College, Pune, IND; 3 Respiratory Medicine, Dr. Bhausaheb Sardesai Talegaon Rural Hospital, Pune, IND

**Keywords:** adenosine deaminase (ada), pleural fluid ada/serum crp ratio, serum crp, serum c-reactive protein, pleural fluid ada, parapneumonic effusion, malignant pleural effusion, tuberculous pleural effusion

## Abstract

Introduction

Pleural effusion is a challenging diagnosis at times, especially due to the overlap of symptoms in effusions of various etiologies. In this study, we aimed to identify if pleural fluid adenosine deaminase (ADA) or serum C-reactive protein (CRP) can be used as an additional novel biomarker for ADA in diagnosing tubercular, parapneumonic, and malignant pleural effusions.

Materials and methods

A prospective, observational, cross-sectional study was conducted on 79 patients diagnosed with tubercular, parapneumonic, or malignant pleural effusion from August 2022 to April 2024 at the Department of Respiratory Medicine at Dr. D. Y. Patil Medical College, Hospital & Research Centre, Pimpri, Pune. The pleural fluid ADA/serum CRP ratio was identified in each group, and analysis was done to compare the ratio in each group. The correlation with pleural fluid ADA was also identified.

Results

A total of 79 patients were enrolled in this study. Out of these patients, 53 (67.1%) were identified as having tubercular pleural effusion, 10 (12.7%) patients had parapneumonic effusion, and 16 (20.3%) had malignant pleural effusion. For malignant effusions, the area under the curve (AUC) using the receiver operating characteristic (ROC) for the ADA/CRP ratio was observed to be 0.862. Sensitivity was 87.50% and specificity was 82.54% at a cut-off value of ≤0.5. The positive predictive value was found to be 56%, and the negative predictive value was found to be 96.3%. For parapneumonic effusions, the AUC using the ROC for the ADA/CRP ratio was observed to be 0.880. Sensitivity was 100% and specificity was 69.57% at a cut-off value of ≤0.67. The positive predictive value was found to be 32.3%, and the negative predictive value was found to be 100%. For tubercular effusions, the AUC using the ROC for the ADA/CRP ratio was observed to be 0.955. Sensitivity was 92.45% and specificity was 88.46% at a cut-off value of >0.54. The positive predictive value was found to be 94.2%, and the negative predictive value was found to be 85.2%. The Pearson correlation coefficient (r) of 0.633 indicates a moderately strong positive linear relationship between ADA and ADA/CRP levels.

Conclusion

The pleural fluid ADA-to-serum CRP ratio can be used as a useful diagnostic tool for differentiating between tubercular, parapneumonic, and malignant pleural effusions. ADA/CRP ratio has added diagnostic value over ADA. In clinically puzzling scenarios, the ADA/CRP ratio can be a cost-effective tool before opting for a more expensive and invasive procedure, which is also often difficult to obtain in resource-limited healthcare settings. More research with a larger sample size is indicated to incorporate the ADA/CRP ratio as an added diagnostic tool along with ADA.

## Introduction

A pleural effusion is a collection of fluid abnormally present in the pleural space, which is mainly caused by excess fluid production and/or poor lymphatic absorption [1]. It is the most prevalent symptom of pleural disease, with causes ranging from cardiac problems and/or systemic inflammatory conditions to cancer. It is challenging to identify the underlying cause of pleural effusions due to the overlap in symptoms caused by various pathologies [2]. The underlying causes of transudative pleural effusions are readily apparent from a thorough history, a physical examination, and a few routine laboratory tests, making the diagnosis quite simple. The majority of exudative pleural effusions, on the other hand, are challenging to diagnose and necessitate a thoracentesis initially, followed by a series of radiological, biochemical, cytological, and microbiological investigations [3-5].

Tubercular, malignant, and parapneumonic effusions are some of the most common causes of exudative effusion that we routinely encounter in our daily clinical practice. Due to the overlap in clinical, radiological, and laboratory profiles, it is challenging to diagnose the exact cause of effusion. Adenosine deaminase (ADA) is routinely used to discriminate between tubercular and non-tubercular effusions at a cutoff value of 40 IU/L [6-8]. Occasionally, there is an overlap in the pleural fluid analysis of tubercular, parapneumonic, and malignant effusions with high ADA levels. In such conditions, it is important to develop a novel biomarker to replace or enhance ADA.

Serum C-reactive protein (CRP) is an acute-phase reactant that can be raised in commonly encountered pulmonary disorders like pneumonia, malignancies, and pulmonary thromboembolism [9]. The cost-effectiveness and easy access to serum CRP are added advantages.

The pleural fluid ADA to serum CRP (ADA/CRP) ratio can be an innovative, cost-effective method for discriminating malignant, parapneumonic, and tuberculous pleural effusions; nevertheless, there is limited and contradictory evidence of its usefulness. The aim of this research is to assess the diagnostic accuracy of the ADA/CRP ratio in distinguishing between tuberculous, parapneumonic, and malignant pleural effusions. Furthermore, we evaluated if the ADA/CRP ratio improved ADA's diagnostic value.

## Materials and methods

A cross-sectional study was conducted in the Department of Respiratory Medicine at Dr. D. Y. Patil Medical College, Hospital & Research Centre, Pimpri, Pune, from November 2022 to April 2024. A total of 79 patients were enrolled in the study. Institutional ethical committee approval was obtained prior to the study (Approval No.: IESC/PGS/2022/62). The study aimed to determine, analyze, and correlate the ratio of ADA and serum CRP in tubercular, parapneumonic, and malignant pleural effusions.

Detailed history-taking and physical examinations were performed on all patients. Diagnostic thoracentesis was carried out post-radiological examination to confirm diagnoses of tubercular, parapneumonic, or malignant pleural effusion. Patients over the age of 18 years, who were confirmed to have tubercular, parapneumonic, or malignant pleural effusions, were enrolled. Exclusion criteria included transudative pleural effusions, other causes of exudative pleural effusion, non-significant pleural effusion (as seen on imaging), hemodynamically unstable patients, and cases of empyema.

A thorough pre-treatment assessment included a detailed history (chief complaints, history of present illness, and comorbidities) and a physical examination. Basic investigations performed on each subject included a complete blood count, liver function tests, renal function tests, serum proteins, serum lactate dehydrogenase (LDH), random blood sugars, glycosylated hemoglobin (HbA1C), and HIV. Serum CRP was additionally tested for all patients. Radiological evaluations included chest X-rays in posteroanterior view, ultrasound of the thorax for fluid quantification, and CT scans where indicated. Diagnostic thoracocentesis was performed on all subjects with aspirable pleural effusion, and pleural fluid was analyzed for total and differential leukocyte count, glucose, proteins, LDH, ADA, CBNAAT (cartridge-based nucleic acid amplification test), gram stain, Ziehl-Neelsen stain, culture & sensitivity, malignant cytology, and acid-fast bacilli (AFB) solid and liquid culture. The exudative nature of the pleural effusion was established using Light’s criteria.

Tubercular pleural effusion was identified by sputum containing AFB, granulomatous lesions with caseation necrosis, characteristic chest radiograph features, pleural fluid ADA ≥ 40 U/L with lymphocyte predominance, a positive CBNAAT test for *Mycobacterium tuberculosis* in pleural fluid (GeneXpert), or pleural fluid positive for *Mycobacterium tuberculosis* in culture or AFB. Malignant pleural effusion was identified by the presence of malignant cells in pleural fluid cytology, pleural biopsy showing malignant infiltrates, or fine needle aspiration cytology/biopsy from lung tumors or lymph nodes confirming carcinoma. Parapneumonic pleural effusion was characterized by an acute febrile illness with purulent sputum, lung infiltrates, or exudates with a high leukocyte count, predominantly neutrophils, high LDH, and low glucose levels in the absence of neoplasia.

Data collected included patient demographics, symptoms and duration, radiological investigations, pleural fluid analyses (ADA, CBNAAT, malignant cytology), serum CRP, and pleural fluid ADA/serum CRP ratio, which were entered into case report forms and later imported into Microsoft Excel (Microsoft Corporation, Redmond, WA) for analysis. Statistical analysis involved calculating the mean, SD, p-values, cut-off values, sensitivity, specificity, positive predictive value, negative predictive value, and area under the receiver operating characteristic (ROC) curve for the three groups (tubercular, parapneumonic, and malignant pleural effusions). Pearson’s correlation coefficient was used to determine the relationship between pleural fluid ADA and the ADA/CRP ratio, and the ANOVA test was used to compare values across different disease types.

## Results

In the context of malignant pleural effusion, the mean pleural fluid ADA level is the lowest among the conditions studied, averaging 17.3 U/L with a relatively small SD of 8.0 U/L. This is in contrast to parapneumonic conditions, where the mean ADA level is significantly higher at 33.5 U/L, accompanied by an SD of 17.3 U/L. Tuberculous conditions exhibit the highest mean ADA level, recorded at 61.3 U/L, with an SD of 18.2 U/L (Table 1).

**Table 1:** Pleural fluid analysis showing mean ± standard deviation for effusions along with p-values. TLC: total leucocyte count; LDH: lactate dehydrogenase; ADA: adenosine deaminase; SD: standard deviation. P-value < 0.001 is significant.

Diagnosis	Malignancy		Parapneumonic		Tubercular		Total		
	Mean	SD	Mean	SD	Mean	SD	Mean	SD	P-value
Pleural fluid TLC	1160.0	850.1	10000.0	0.0	1608.3	1139.7	2579.7	3019.7	<0.001
Neutrophils	20.3	11.2	66.5	9.4	16.3	12.6	23.5	20.4	<0.001
Lymphocytes	65.6	14.1	23.0	7.5	75.6	12.7	66.9	21.2	<0.001
Mesothelial cells	14.1	7.8	9.5	4.4	7.9	2.7	9.4	5.0	<0.001
Sugar	88.1	55.9	62.2	41.0	96.7	52.8	90.6	52.7	0.162
Protein	5.4	1.5	4.6	0.6	5.3	0.7	5.2	0.9	0.063
LDH	468.4	251.9	414.5	183.9	415.4	254.1	426.1	244.1	0.744
ADA	17.3	8.0	33.5	17.3	61.3	18.2	48.9	24.6	<0.001

Regarding serum CRP levels, malignant pleural effusion shows a mean level of 54.8 mg/L with an SD of 30.0 mg/L. This contrasts sharply with parapneumonic effusions, which present a much higher mean serum CRP level of 162.1 mg/L and a substantial SD of 139.2 mg/L. In tuberculous effusions, the mean serum CRP level is the lowest at 41.5 mg/L, with an SD of 35.9 mg/L (Table 2).

**Table 2:** Mean ± standard deviation for serum CRP values for pleural effusions. CRP: C-reactive protein.

Diagnosis		Serum CRP
Malignancy	Mean	54.8
	SD	30.0
Parapneumonic	Mean	162.1
	SD	139.2
Tubercular	Mean	41.5
	SD	35.9

The ADA/CRP ratio further distinguishes these conditions. For malignant pleural effusion, the mean ADA/CRP ratio is 0.4 with an SD of 0.5. In parapneumonic conditions, the mean ratio is slightly lower at 0.3, with an SD of 0.2. In contrast, tuberculous conditions exhibit a markedly higher mean ADA/CRP ratio of 2.1, with an SD of 1.0 (Table 3).

**Table 3:** Mean ± SD and p-value for ADA/CRP ratio for pleural effusions. ADA: adenosine deaminase; CRP: C-reactive protein. P-value < 0.001 is significant.

Diagnosis	ADA/CRP ratio		P-value
Malignancy	Mean	0.4	<0.001
	SD	0.5	
Parapneumonic	Mean	0.3	
	SD	0.2	
Tubercular	Mean	2.1	
	SD	1.0	

A strong positive correlation is noted between pleural fluid ADA levels and the pleural fluid ADA/serum CRP ratio, with Pearson's correlation coefficient (r) calculated at 0.633. This indicates a significant positive association between these two variables, suggesting that as ADA levels increase, the ADA/CRP ratio also tends to rise proportionally.

**Figure 1:**
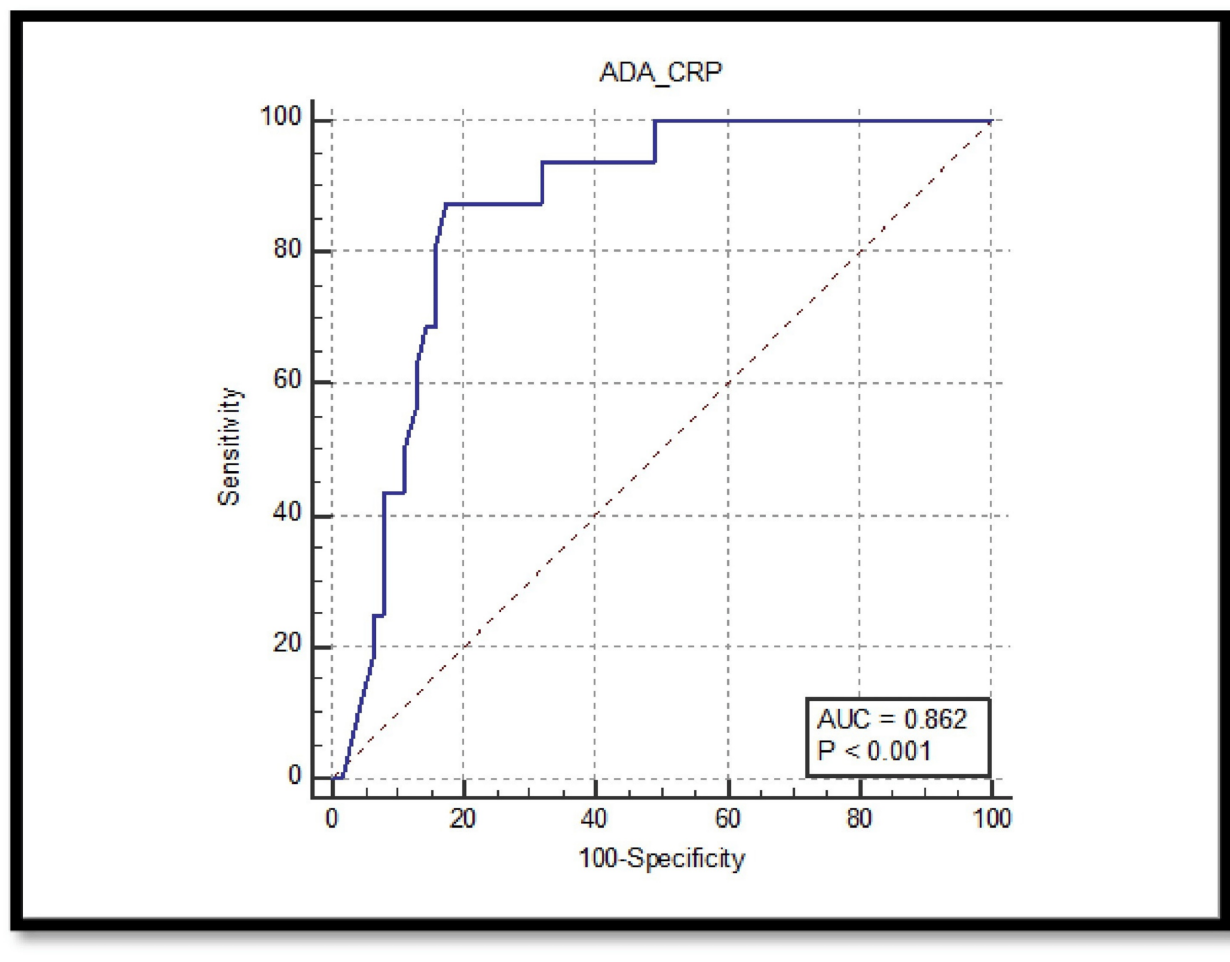
ROC for ADA/CRP ratio for malignant effusions with area under the curve of 0.862 and p-value < 0.001. ADA: adenosine deaminase; CRP: C-reactive protein; AUC: area under the curve; ROC: receiver operating characteristic.

When evaluating the diagnostic performance of the ADA/CRP ratio through the ROC curve analysis, several critical observations emerge. For malignant effusions, the area under the curve (AUC) is 0.862. The sensitivity at a cut-off value of ≤0.5 is 87.50%, and the specificity is 82.54%. This cut-off yields a positive predictive value (PPV) of 56% and a negative predictive value (NPV) of 96.3%, indicating the ratio’s effectiveness in distinguishing malignant effusions (Figure 1).

**Figure 2:**
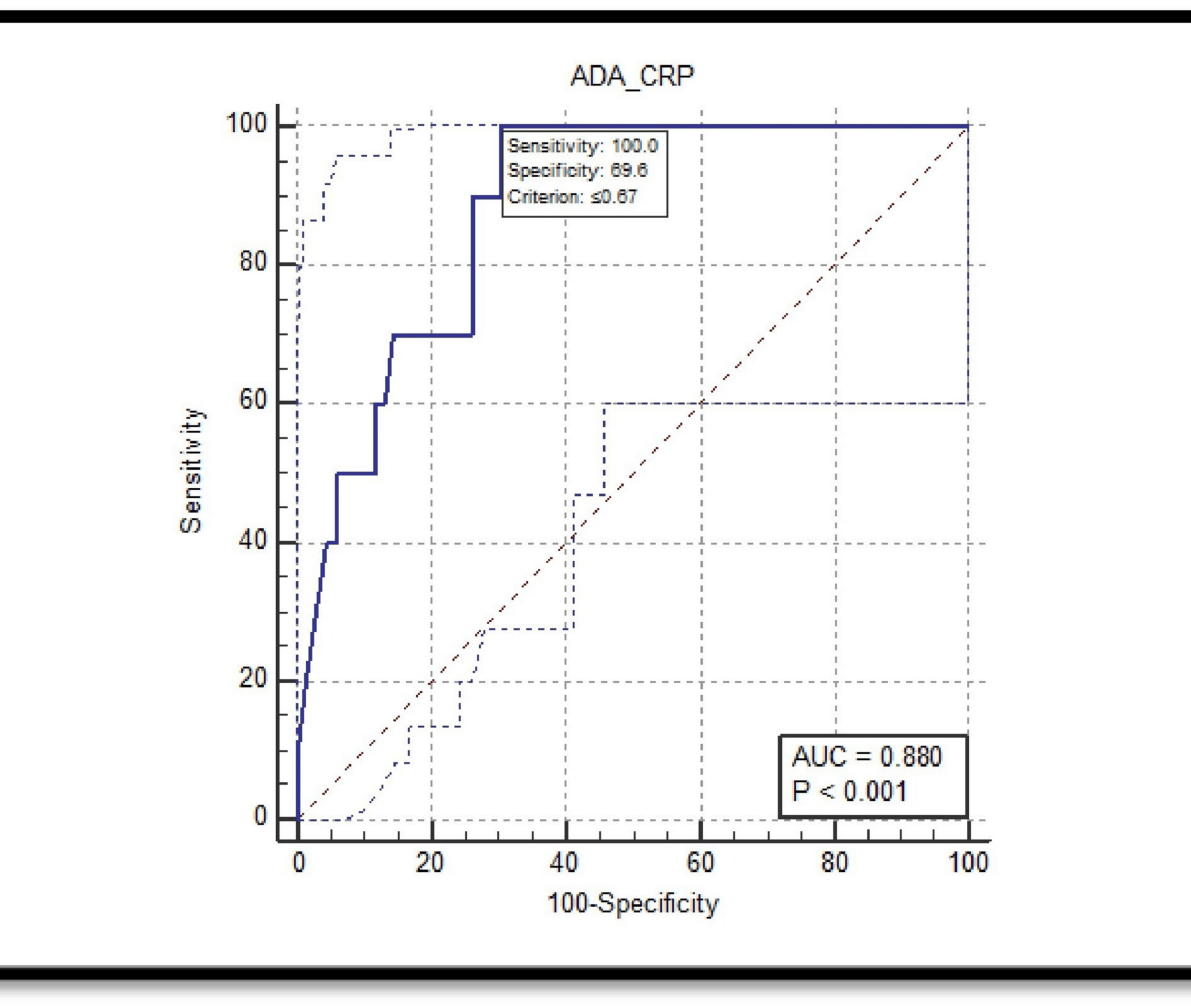
ROC for ADA/CRP ratio for parapneumonic effusions with area under the curve of 0.880 and p-value < 0.001. ADA: adenosine deaminase; CRP: C-reactive protein; AUC: area under the curve; ROC: receiver operating characteristic.

For parapneumonic effusions, the ROC curve analysis revealed an AUC of 0.880. The sensitivity at a cut-off value of ≤0.67 is 100%, while the specificity is 69.57%. The PPV at this cut-off is 32.3%, and the NPV is 100%, demonstrating high sensitivity but moderate specificity for identifying parapneumonic effusions (Figure 2).

**Figure 3:**
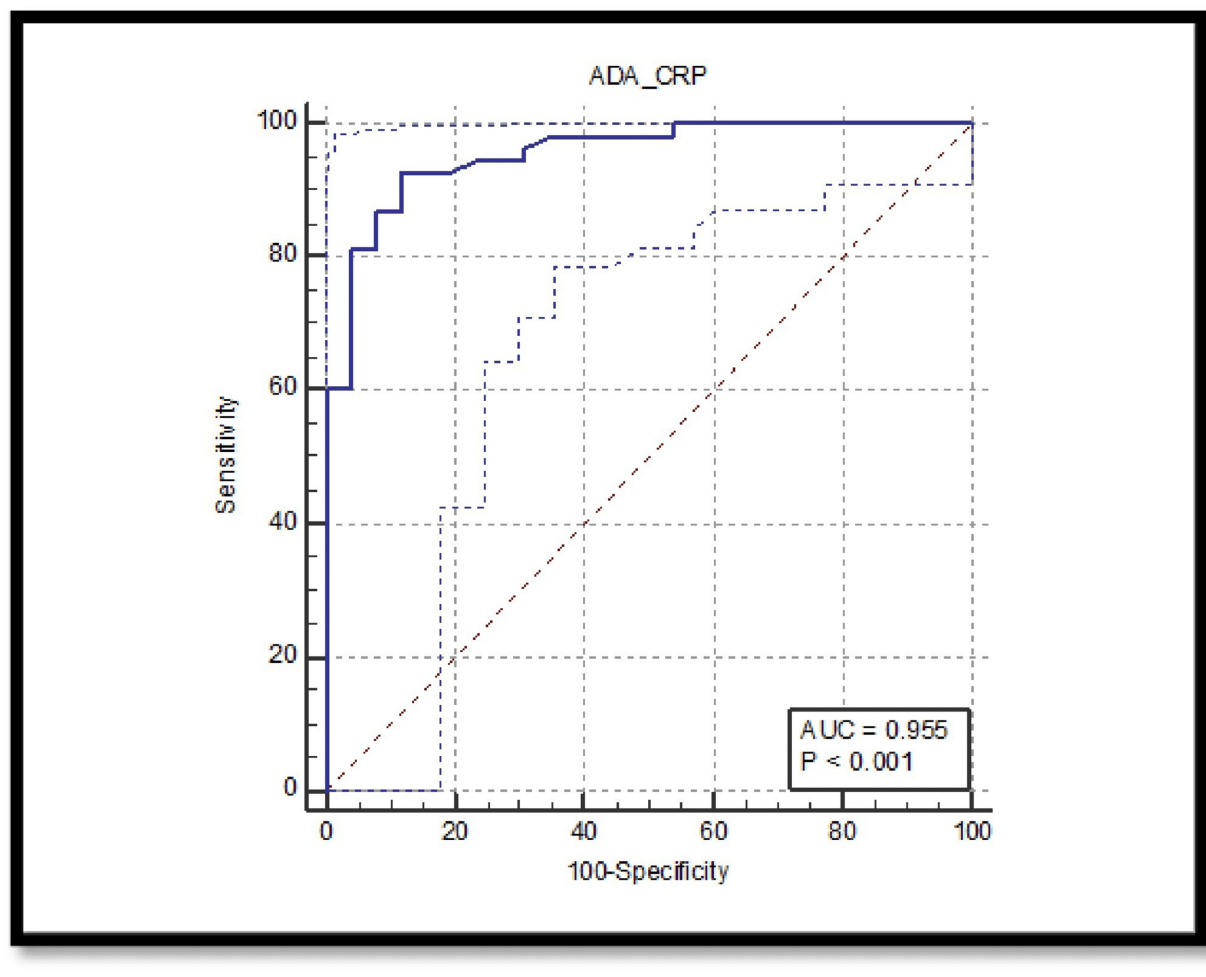
ROC for ADA/CRP ratio for tubercular effusions with area under the curve of 0.955 and p-value < 0.001. ADA: adenosine deaminase; CRP: C-reactive protein; AUC: area under the curve; ROC: receiver operating characteristic.

Tuberculous effusions show the highest diagnostic accuracy with an AUC of 0.955. At a cut-off value of >0.54, the sensitivity is 92.45% and the specificity is 88.46%. The PPV is 94.2%, and the NPV is 85.2%, highlighting the ADA/CRP ratio’s strong diagnostic power for tuberculous effusions (Figure 3).

## Discussion

The ADA/CRP ratio has emerged as a promising biomarker for diagnosing malignant effusions [10]. In our study, the ROC analysis demonstrated an AUC of 0.862, indicating a strong ability to differentiate malignant from non-malignant effusions. With a sensitivity of 87.50% and specificity of 82.54% at a cut-off value of ≤0.5, this ratio is highly effective in detecting malignant effusions while correctly identifying non-malignant cases. This aligns with the findings of Venkatesh et al. [11], who observed significant differences in ADA/CRP ratios between malignant pleural effusion (MPE) and tuberculous pleural effusion (TPE), with mean ratios of 1.36 ± 1.28 and 6.96 ± 7.32, respectively (p = 0.0006). Their ROC analysis suggested a slightly different cut-off (≤1.2) for distinguishing MPE from TPE, with a sensitivity of 78.95% and specificity of 83.33%, reinforcing our findings on the utility of the ADA/CRP ratio in clinical practice.

The high NPV of 96.3% in our study indicates that a ratio above the threshold reliably excludes malignancy, providing significant reassurance in clinical settings and potentially reducing the need for more invasive diagnostic procedures. However, the PPV of 56% reflects a moderate probability of malignancy when the ratio is positive, suggesting the necessity for additional confirmatory tests. This moderate PPV is a reminder of the complex nature of diagnosing malignant effusions, where overlapping clinical and biochemical features with other conditions can lead to false positives. The study by Rabbi et al. [12] supports this perspective, highlighting that while the pleural fluid ADA level and the ADA/CRP ratio were significantly higher in TPE compared to MPE, there was no significant difference in serum CRP levels between these groups. Their findings suggest a cut-off value of >1.25 for diagnosing TPE, with a sensitivity of 93.8% and specificity of 85.2%, further emphasizing the importance of context-specific cut-off values for different effusions.

Overall, our findings underscore the ADA/CRP ratio's utility as a valuable biomarker for distinguishing between malignant and non-malignant effusions, contributing significantly to diagnostic accuracy in clinical practice. However, due to the moderate PPV, the ADA/CRP ratio should be used in conjunction with other diagnostic tools to confirm malignancy.

For parapneumonic effusions, the ADA/CRP ratio also shows strong diagnostic potential. Our study found an AUC of 0.880, indicating good overall discriminative ability. At a cut-off value of ≤0.67, the ratio demonstrated perfect sensitivity (100%), meaning it correctly identified all patients with parapneumonic effusions. This makes the ADA/CRP ratio a highly effective tool for ruling out parapneumonic effusions when the test is negative, aligning with the findings of Lee et al. [10]. Their research indicated that the ADA/CRP ratio had high diagnostic accuracy (AUC of 0.93) in differentiating tuberculous from parapneumonic effusions, particularly in cases with predominant neutrophils and elevated ADA levels.

However, the specificity of 69.57% in our study suggests that the ADA/CRP ratio correctly identifies about 70% of patients without parapneumonic effusions but also produces a substantial number of false positives. This is reflected in the PPV of 32.3%, indicating that among those identified as positive by the ADA/CRP ratio, approximately one-third had parapneumonic effusions. Such a low PPV necessitates a cautious interpretation of positive results and emphasizes the need for additional diagnostic evaluation or testing to confirm the diagnosis. Conversely, the NPV of 100% in our study provides robust confidence in excluding parapneumonic effusions when the ratio is below the threshold, streamlining the diagnostic process and helping clinicians focus on alternative diagnoses. These findings are consistent with Gowtham et al. [13], who reported high sensitivity (97.6%) and specificity (92.3%) for differentiating tuberculous from parapneumonic effusions using the ADA/CRP ratio, with an AUC of 0.988.

Thus, while the ADA/CRP ratio is a valuable initial screening tool for parapneumonic effusions due to its high sensitivity and excellent NPV, the moderate specificity and low PPV highlight the need for integrating this ratio with other diagnostic modalities to achieve accurate and comprehensive patient assessment.

In diagnosing tubercular effusions, the ADA/CRP ratio demonstrates exceptional performance. Our study shows an AUC of 0.955, indicating near-perfect discriminatory ability. At a cut-off value of >0.54, the ratio achieved high sensitivity (92.45%) and specificity (88.46%), making it a reliable indicator for identifying tubercular effusions. This finding is supported by Rabbi et al. [12], who reported an AUC of 0.94 with a cut-off value of >1.25, yielding a sensitivity of 93.8% and specificity of 85.2% for diagnosing TPE. The high PPV of 94.2% in our study suggests that most patients with a ratio above the cut-off value likely have tubercular effusions, facilitating confident diagnosis and timely treatment initiation. The NPV of 85.2% also supports the ratio's efficacy in ruling out the condition when the value is at or below the threshold, ensuring that patients are not unnecessarily subjected to treatment for tuberculosis.

These findings align with those of Venkatesh et al. [11], who found significant differences in the ADA/CRP ratio between TPE and MPE, with higher ratios in TPE. Additionally, Lee et al. [10] highlighted the ADA/CRP ratio's high diagnostic accuracy in differentiating TPE from parapneumonic effusion, especially when the cut-off value was set at 5.62, resulting in a sensitivity of 89% and specificity of 88%. These studies collectively underscore the ADA/CRP ratio's value in distinguishing tubercular effusions in various clinical scenarios. In settings where tuberculosis is prevalent, the ADA/CRP ratio serves as a cost-effective and accessible diagnostic tool, augmenting clinicians' ability to diagnose and manage tubercular effusions effectively. The high AUC and robust sensitivity and specificity values across different studies reinforce its role as a valuable biomarker in clinical practice.

The study revealed significant variability in the standard deviation values. This variability can be attributable to multiple reasons. Biomarker levels vary a lot when there are conditions with different levels of severity and underlying pathology, like tuberculous, parapneumonic, and malignant pleural effusions. Moreover, hereditary factors and variations in metabolic and immunological responses contribute to the wide range of observed values for ADA and CRP levels, adding to individual heterogeneity. Patients at different stages of disease progression, with varying illness presentations and consequences, also demonstrate a wide range of ADA and CRP values, contributing to the total variability [14,15]. Also, having more than one illness at the same time and having different reactions to current treatments can greatly change these biomarker levels, which is why the standard deviations are so large. The study's limited sample size of 79 individuals and its single-center methodology results in extreme values.

While the study provides valuable insights, the study also has a number of limitations. The limited sample size of 79 patients and the fact that the study was conducted at a single center restrict the capacity to apply the findings to a broader population. The presence of selection bias in the study may be attributed to the inclusion criteria, which could have led to the exclusion of atypical cases and consequently influenced the outcomes. Moreover, the requirement for comprehensive demographic and clinical diversity in the study sample limits the generalizability of the results to various patient cohorts. Being a cross-sectional, observational study, it lacks the ability to consider changes in ADA and CRP levels over time or the advancement of illnesses, which makes it difficult to establish a cause-and-effect relationship. The variability in laboratory measurements of ADA and CRP may potentially affect the repeatability and reliability of the ADA/CRP ratio. The study failed to consider any confounding factors, such as comorbidities or concurrent infections, that may affect ADA and CRP levels, perhaps resulting in a misinterpretation of the diagnostic usefulness of the ratio. Moreover, the relatively moderate PPV of 56% for malignancy indicates that a positive ADA/CRP ratio does not provide robust confirmation of malignancy. Therefore, additional diagnostic tests are required. The presence of similar amounts of ADA and CRP in various types of effusions makes it difficult to differentiate between them and reduces the usefulness of the ratio as a unique biomarker. Further validation in various clinical situations and populations is necessary to verify these findings. The study's exclusive focus on tubercular, parapneumonic, and malignant effusions disregards other forms of exudative pleural effusions, hence restricting its wider relevance. Future research should incorporate bigger, multi-center studies to enhance the generalizability of the findings. Longitudinal studies should be conducted to evaluate changes in biomarkers over time. Additionally, adjustments for confounding variables should be made to isolate the underlying influence of ADA and CRP levels. In addition, investigating alternative biomarkers could improve the precision of diagnosis in cases where ADA/CRP findings are unclear. To improve the clinical usefulness of the ADA/CRP ratio in diagnosing pleural effusions, it is necessary to address these limitations.

## Conclusions

This study presents evidence that the ratio of pleural fluid ADA to serum CRP is a potentially valuable biomarker for differentiating tuberculous effusions from parapneumonic and malignant pleural effusions. The results suggest that the ADA/CRP ratio is a highly accurate diagnostic tool, especially for identifying tuberculous pleural effusions. Tuberculous effusions had the highest mean ratio and the most robust AUC in the ROC study. Although the ADA/CRP ratio has the potential to identify malignant and parapneumonic effusions, its PPV varies. Therefore, it is recommended to employ this ratio along with other diagnostic tools to confirm the underlying etiology of pleural effusion. The study emphasizes the importance of taking into account the variability in patient presentations and underlying diseases, as these factors contribute to the significant variations reported in biomarker levels. The wide range of variations highlights the complex nature of employing ADA and CRP as independent diagnostic tools, indicating that the ADA/CRP ratio is most effectively utilized as a component of a comprehensive diagnostic approach. The strong correlation between levels of ADA in pleural fluid and the ADA/CRP ratio further supports the potential usefulness of this ratio in clinical settings. It provides a cost-effective and easily accessible method to assist in the differential diagnosis of pleural effusions. Nevertheless, the study's limited sample size and the impact of confounding variables necessitate bigger, multi-center research to validate these findings and enhance the precision of the diagnostic cut-off values. In summary, the ADA/CRP ratio has great potential as an additional biomarker for assessing pleural effusions. Future research should focus on determining more accurate thresholds and incorporating this ratio into wider diagnostic frameworks to improve its clinical usefulness and dependability.
